# Monitoring Environmental Exposures: Now It’s Personal

**DOI:** 10.1289/ehp.114-a528

**Published:** 2006-09

**Authors:** Charles W. Schmidt

Most diseases are thought to arise from the combined effects of genes and the environment. While great strides have been made in our understanding of human genetics, the contribution of environmental exposures to disease remains poorly understood. This lack of understanding impedes real progress in identifying genetically susceptible people whose responses to environmental agents are severe or unique relative to the general population. If identified, targeted prevention and treatment strategies might be applied to these groups, with potentially lifesaving results. Without more personal exposure information, however, scientists have a limited ability to identify pollution-susceptibility genes that elevate disease risk.

This mismatch between exposure and genetic research slows the identification of environmental factors that—if altered or removed—could even prevent some diseases from occurring in the first place. “Genes aren’t modifiable,” explains Frederica Perera, a professor of environmental health sciences at the Mailman School of Public Health of Columbia University. “So, these environmental components in diet, food, water, and air are the only ones we can act on for disease prevention.”

Exposure is, at a core level, the instance of environmental stimuli such as chemicals, infectious agents, diet, and lifestyle factors interacting with the human body. But studying human exposures poses difficult challenges. Scientists studying the effects of chemical pollutants can’t ethically dose people, so they often base their dose–response estimates on animal models. Further, they base their estimates of human exposure on indirect measures taken from a person’s home or work environment, along with assumptions about individual behaviors that influence the risk of coming into contact with a given pollutant. In a typical study, epidemiologists might use census figures, questionnaires, and general environmental monitoring data (such as samples of water from a household tap or soil in a schoolyard) to estimate how much of a given chemical an individual has come into contact with, and for how long.

But while these studies approximate population-level exposures, they provide little information about real exposures to individual people. And because of this, scientists know little about how specific pollutants—particularly in combination with each other, with diet, and with physical activity—affect any individual’s response to the pollutant.

With a clear need for progress, exposure assessment has recently come under the spotlight. This year, the NIEHS launched the Exposure Biology Program (EBP), a four-year effort with two overarching goals: to improve exposure assessment technology, and to identify biomarkers for common pathogenic mechanisms that reflect the human response to environmental agents. Such biomarkers could include changes in metabolites, proteins, or DNA that reflect the individual’s genetic susceptibility to environmental harm.

“Right now we don’t really understand how exposure levels translate to human health risk, so our goal is to fill that gap,” says EBP coordinator Brenda Weis, a senior scientific advisor at NIEHS. “We need to get a better measure of exposure at the point of human contact, and we need to integrate those measures with biological response measures derived from samples taken directly from exposed people. So, this is a more ‘medical’ approach to exposure assessment—in the sense that measures are taken on a personal level—rather than the broader, ecological approaches we’ve been using so far.”

## The Genes and Environment Initiative

The EBP is part of the larger DHHS Genes and Environment Initiative (GEI), which was established on 8 February 2006. This initiative links the EBP with a complementary genome association program, led by the National Human Genome Research Institute (NHGRI), which is dedicated to finding genetic risk factors for environmental diseases.

NIEHS director David Schwartz and NHGRI director Francis Collins initially hatched the idea for a joint collaboration two years ago. At first, the two scientists proposed assembling a vast cohort of 1 million Americans for a 10-year prospective study of gene–environment interactions in health. However, that plan has not yet materialized, because of its high anticipated cost—estimated at up to $3 billion—and also because experts at both agencies felt that policies for genetic privacy and other social issues needed to be further developed before such a large study could be undertaken.

The GEI was therefore proposed as a more limited effort encompassing smaller population studies and fewer diseases. “Schwartz will head the exposure piece while Collins will head up the GEI’s genome association studies,” Weis explains. “The idea was to unite our mutual expertise—we worked for a long time on the concept, and we wound up with forty million dollars in annual funding for four years.”

NIH director Elias Zerhouni said of the GEI upon its launch that “[this initiative] would not have been possible a year or two ago.” Recent years have given rise to medical and computational advances that make detailed studies of gene–environment interactions increasingly more feasible. The cost of genotyping, for instance, meaning the identification of specific genetic variants at one or more loci in an individual, has dropped by more than 100-fold, making the initiative’s genome association component more affordable. Environmental sensors that monitor individual pollution exposures, physical activity patterns, and corresponding physiological responses have also met with rapid progress, bringing goals for personalized exposure assessment within reach.

Through the EBP, Weis says, the NIEHS will develop technologies that produce more precise measures of exposure, and with that, better biomarkers of physiological response. The NHGRI, meanwhile, plans to apply those biomarkers toward genome association studies in specific illnesses, such as cancer, respiratory ailments, and heart disease. “We’ll also offer input on the [biomarkers of] exposure that the scientists select,” says Teri Manolio, a senior advisor at the NHGRI managing GEI-related activities. “We need to be sure they relate to genetics as well as environment.” Through the combined efforts of both institutes, adds Weis, the GEI will accelerate progress on gene–environment research and lay the technical and social foundations for larger studies in the future.

A step forward on the NIEHS’s exposure assessment agenda occurred on 16–17 May 2006, when academic, government, and industry experts convened in Greensboro, North Carolina, for the NIH Exposure Biology Workshop, hosted by the NIEHS and the NHGRI. At the workshop, experts deliberated the pitfalls and promises of exposure assessment, and produced a series of recommendations for optimizing sensor technology and biomarker development.

The meeting was remarkably interdisciplinary, participants say. “I’m used to going to meetings where I know everyone,” says Martyn Smith, a professor of toxicology at the University of California, Berkeley, who was in attendance. “But I didn’t know many of the people there, and I think that’s a good thing. There needs to be more interaction between the engineers who design exposure instruments and we in environmental health who apply them in the field.”

## New Sensations

Among the tools used to monitor exposures now are laser-based sensors that assess population exposures to industrial stack emissions, wearable dosimeters that measure chemicals and radiation (generally in occupational settings), accelerometers that measure physical activity, and biosensors that can detect specific analytes in the body.

New technologies to assess personal exposures are being conceptualized, but developments are moving slowly. A research article by Weis and colleagues published in the July 2005 issue of *EHP* outlines current, ongoing efforts. The article describes promising applications that link geographic information system (GIS) technology with fate and transport models to derive individual exposure metrics for pesticides, drinking water contaminants, and air pollutants. The article further reports that researchers have used GIS with Global Positioning System (GPS) technology to define activity patterns that could conceivably be linked to environmental data for exposure assessment. However, sources contacted for the present article were unaware of any existing GIS applications for environmental epidemiology research.

Some of the most exciting sensing opportunities have come from military and biomedical research. The military has produced sensors that detect biological warfare agents such as anthrax down to the level of a single spore. Medical researchers, meanwhile, have developed biosensors that detect isolated binding events in single cells, in addition to variables like heart rate, respiratory function, and changes in enzyme levels. “We have a glaring, obvious opportunity to adapt these tools for environmental exposure assessment,” says David Walt, a professor of chemistry at Tufts University. “We can use them to monitor the ambient environment as well as personal exposure. We now have the capability to take all those measurements and link them together.”

Future sensing opportunities are nothing short of extraordinary. Subjects could be injected with nanoscale biosensors that light up when they detect exposure-induced molecular changes. “After a period of time, a blood sample could be taken, the biosensors could be fished out, and you’d see precisely what you’d been exposed to,” Walt speculates. “The techniques are available now, but they haven’t been addressed to environmental exposure.” Some biosensors outfitted with microtransducers could relay information about cellular changes with electronic signals, he adds. “And in those cases you might not even have to take the blood sample—you could read an integrated signal of all the particles in an interrogation zone right through the skin.”

William Haskell, a professor at the Stanford University School of Medicine, proposes to create a portable device that measures physical activity, pollution exposure, and other variables simultaneously in real time. The sensing instruments, he suggests, could be loaded onto a GPS-enabled cell phone into which subjects could record their diet and other pertinent information using the keypad.

Haskell is collaborating on the concept with Stephen Intille, a research scientist at the Massachusetts Institute of Technology who’s been developing wireless motion sensors to measure a variety of human behaviors. Their immediate goal is to create cell phone–adaptable software that distinguishes whether someone is walking, riding a bike, working in the yard, or doing something else, all according to how their physical motions affect heart rate, and how they accelerate and decelerate over time. That capability, along with GPS and sensors that detect chemicals in the cell phone’s immediate vicinity, could give scientists unprecedented access to the locations, activities, diets, and chemical exposures of individuals in a given cohort.

What would scientists do with that information? “Say you were a pediatrician in Los Angeles,” Haskell proposes, “and you wanted to understand what triggers asthma in kids. With these more advanced technologies you could plot their activity patterns against pollution exposure levels. That would allow you to draw a personal profile that, upon being transmitted by the cell phone, could be automatically downloaded onto a server. We could use that information to determine the specific components in air pollution that trigger asthma in high-risk individuals. We have the technical capability to do this now; it’s just a matter of pulling the resources together for this particular purpose.”

Future sensors, scientists say, need to be even faster, cheaper, and more portable than they are now. Participants at the May workshop called for more collaboration with sensor developers, and clear lines of communication regarding the specific measurements needed to move exposure assessment forward. Along these lines, “high-benefit” opportunities are expected in lab-on-a-chip platforms (which analyze pollutants in the field as opposed to in the laboratory) and multiplexed sensors that monitor multiple pollutants simultaneously.

## Fingering the Biomarkers

The chief goal, of course, is to link exposure measurements to biomarkers that reflect a physiological response. John Groopman, who chairs the Department of Environmental Health Sciences at the Johns Hopkins Bloomberg School of Public Health, argues that scientists should first seek biomarkers for diseases with known links to the environment. “And certainly a number of lung-related effects fulfill that criteria,” he says.

Weis agrees, and points out that the EBP’s first project, an airway disease project launched in 2006, will investigate the impacts of cigarette smoke, ozone, cockroach dander, and lipopolysaccharide (found on the cell walls of Gram-negative bacteria) on two processes related to respiratory disease: inflammation and oxidative stress. “It would be really informative to find a panel of biomarkers that’s specific to each of these agents,” she says.

Scientists will begin by searching for putative biomarkers in animals that can be further investigated in people going about their daily business who will be outfitted with personal exposure monitors. Another goal is to use exposure agents to find biomarkers that distinguish different types of asthma—for instance, using an immune response triggered by cockroach dander to distinguish allergenic asthma from nonallergenic asthmatic reactions caused by other environmental agents. “People with different types of asthma could be treated with therapies tailored to their type of asthma,” Weis explains. “But we need to understand the mechanistic differences between the various manifestations of the disease. We want to better understand those mechanisms so we can improve treatment and prevention options.”

Focusing on inflammation and oxidative stress makes additional sense because both pathways are also common to other ailments, including cancer and heart disease. But scientists studying these pathways must consider their normal equilibrium, stresses Martha Monick, a research scientist at the University of Iowa. Inflammation and oxidative stress normally protect the body from pathogens and are only toxic when overstimulated, she says, and that natural variability should be considered during the exposure assessment. Moreover, she adds, the inflammatory response to environmental factors can, in some cases, merely exacerbate preexisting conditions with nonenvironmental origins. For instance, atherosclerosis, a progressive vascular disease with an underlying basis in genetics, can be aggravated by exposure to cigarette smoke and air pollutants such as particulate matter. “So it would be helpful to have biomarkers for exposures that prolong a disease in addition to those that might cause it,” she says.

EBP workshop participants stressed that metabolomic, proteomic, and genomic methods offer new opportunities for biomarker development. DNA and protein adducts in particular could provide windows into the body’s long-term “memory” of previous exposures, they suggested. Metabolite profiles, meanwhile, could offer shorter-term measures of environmental response. Perera explains, “The DNA and protein adducts in peripheral blood—at least for polycyclic aromatic hydrocarbons in settings of constant exposure—have lifetimes on the order of several months. The metabolite profiles are likely to be more short-lived.”

Bruce Hammock, a professor of entomology and toxicology at the University of California, Davis, says metabolomics—like the other “omic” sciences—has benefited from economies of scale that have dramatically reduced analytical costs. He says the growing capacity to measure dozens, even hundreds, of metabolites simultaneously opens doors for two types of research.

The first involves an unbiased interrogation of all measurable metabolites induced by a particular environmental exposure. This global approach could yield biomarkers linking the exposure to entirely new and perhaps unexpected diseases. “Say we’re studying air pollution and asthma,” Smith says. “Recent evidence shows airborne particulates play an important role in heart disease, so if you focus just on asthma, then you might miss that additional connection.”

With the second type of research, scientists could investigate specific metabolic pathways linked to a specific ailment—for instance, air pollution–induced inflammation and heart disease. This hypothesis-driven method would allow scientists to backtrack along the exposure response to biomarkers that offer clues to underlying physiology.

Another point to consider, Groopman adds, is that mass spectrometry is 1,000-fold more sensitive today than it was 15 to 20 years ago, which makes it possible to detect ever-smaller concentrations of chemicals in a given sample. Scientists who previously might have detected a pollutant in the blood of just a few subjects might now find it at very low levels in many more, he says. And by linking those pollutant analyses with corresponding gene, protein, and metabolite profiles in exposed people, it might finally be possible to assess the health effects of low-level chemical mixtures, which has long been a holy grail for environmental health scientists.

## Social Concerns and Regulatory Implications

As it stands now, the coming era of personal exposure assessment is poised for takeoff. Dialogue, collaboration, and the promise of funding have brought a preliminary vision into focus, and the next several years will see increasingly refined technologies applied to the field. But exposure assessment’s new direction also raises some difficult questions. In the not-too-distant future, thousands of people could be enrolled in highly invasive studies, their pollutant levels, diets, movements, and physical activities all monitored in real time with wireless sensors linked to growing databases. Kathy Hudson, who directs the Genetics and Public Policy Center in Washington, DC, says, “We’re talking about peering into people’s private lives in a very profound way, so the question is whether they will accept that intrusion into their personal space.”

Who would participate in these studies? “Certainly highly motivated people,” Weis answers. “Parents who have kids with asthma would be more likely to enroll. We’re trying to address this issue now—we know data collection devices would have to be portable and easy to carry around.”

Linda Sheldon, the acting director for human exposure and atmospheric sciences at the EPA, suggests researchers need to reach out to prospective communities and listen to their concerns. “You have to work with community groups and help them understand how this knowledge can benefit them and society in general,” she says. “And you really need to focus on people who are interested in the environment and its impacts on their health and the health of their kids.”

Beyond the recruitment challenges, gene–environment research in general raises questions about genetic privacy. Could subjects be harmed by relinquishing genetic information to the public? Hudson says there’s little evidence to suggest individuals are more concerned about disclosing their genotypes than they are about disclosing the dietary and lifestyle factors already used in health research. But she concedes the GEI and future larger-scale projects are breaking new ground in this area.

“For all these data to be used to expand our knowledge of disease, they have to be widely available,” Hudson says. “So, we have to consider the kinds of access and uses we want to prevent. On the one hand, we want that [gene–environment] information to be available, but on the other hand we don’t want those who give of themselves to research to put themselves at risk.”

Hudson says researchers also need to consider the consequences of their findings. For instance, she asks, how and at what point are correlations that link exposure and environmental disease relayed back to the public? Who pays for the removal of confirmed risk factors and the treatment of identified health conditions? What obligations do researchers have to communicate findings to their cohorts? “You really have to think carefully about all these outcomes,” Hudson says.

Over time, findings that emerge from exposure assessment’s growing alliance with genetics could produce social benefits by improved regulation. Sheldon says that whereas the EPA built its success on addressing “the big, obvious pollution problems,” it is now facing more subtle issues such as understanding and managing health risks from low-level pollutant mixtures to which most people are exposed.

“We need more precise tools to understand the problems we face now,” Hudson says. “Once these chemicals pass into the body, the effects may be different in different people—we need to get a better handle on the sequence of events that ultimately leads to a health outcome.”

Ultimately, this could help us improve the safety factors we use in risk assessment as well as truly understand the risks. “The findings may not translate immediately,” says Sheldon, “but as the science builds up it will have an impact.”

## Moving Forward

As the EBP moves forward, a GEI Subcommittee on Exposure Biology made up of experts from throughout the NIH is meeting on a biweekly basis in Bethesda to discuss priorities and progress. According to Weis, discussions emphasize fiscal year 2007 research initiatives in environmental sensors and biomarker development, which will address assessment needs for environmental pollutants, diet, physical activity, and even psychosocial stressors. Meanwhile, a counterpart GEI Genetics Subcommittee is addressing ongoing issues related to the initiative’s genome association studies. Both subcommittees are open to all NIH staff.

Summing up, Perera suggests that emerging advances in exposure assessment will allow researchers to make better use of population resources.

“Everyone’s excited about moving on to this next level,” she says. “Researchers like us are eager to do more with our cohorts, and to find new molecular links between exposure and disease. This is all really about identifying preventable environmental exposures and their role in diseases that are extremely prevalent in our society.”

## Figures and Tables

**Figure:**
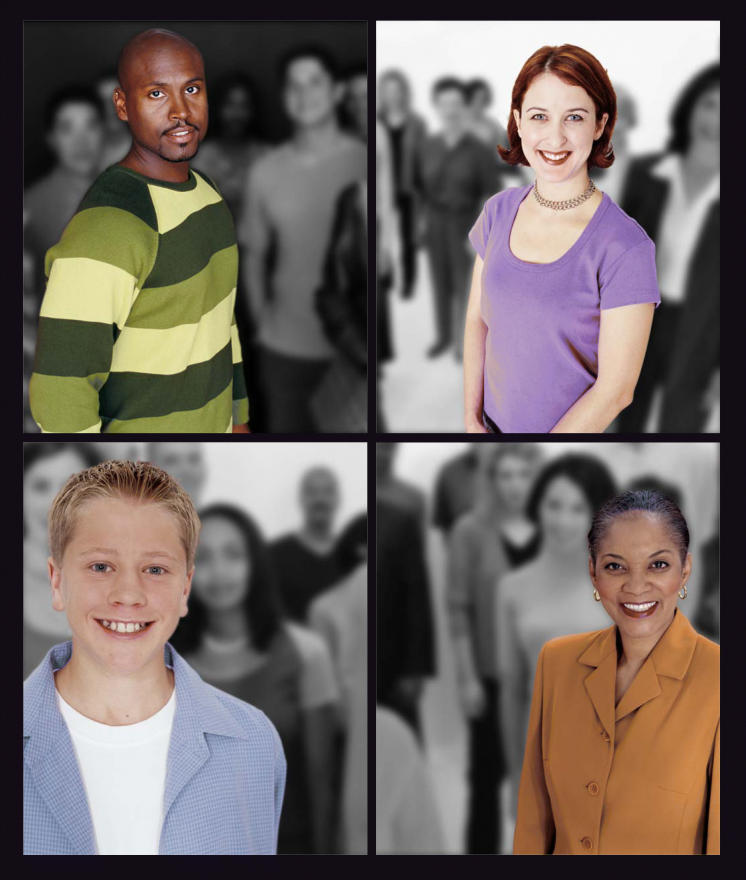


**Figure:**
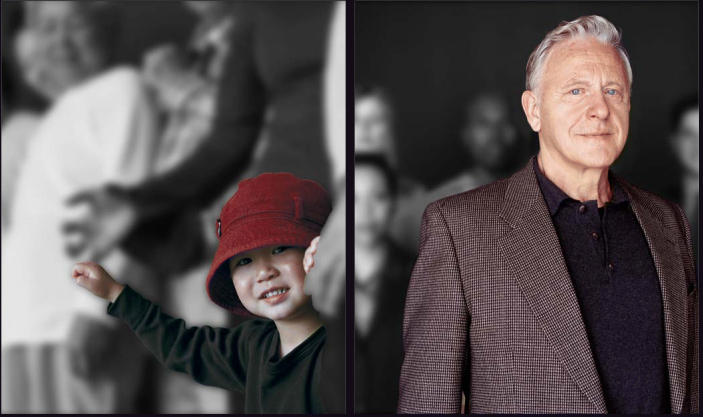


**Figure:**
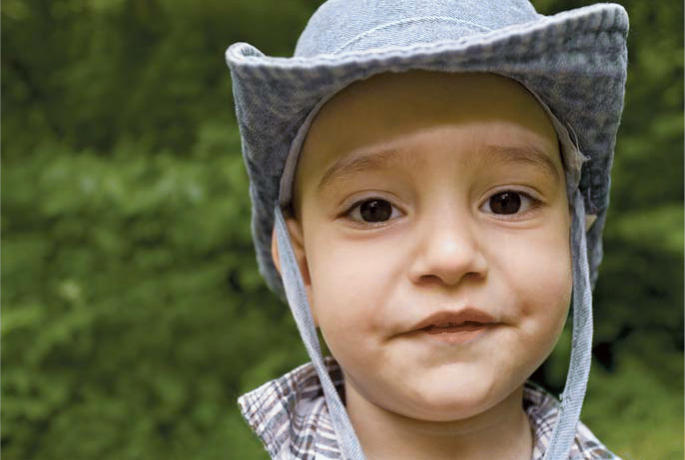
Outside impacts on inside systems Measuring responses to factors such as sun exposure and allergies may provide important information about the health impact of environmental stress.

**Figure:**
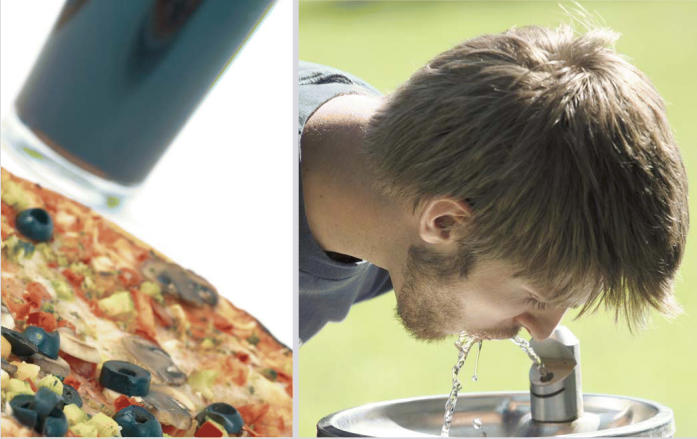
It’s a combo deal Any given person has a highly unique suite of exposures, making individual-level exposure assessment a must for teasing out how factors such as diet and physical activity influence people’s responses to particular pollutants.

**Figure:**
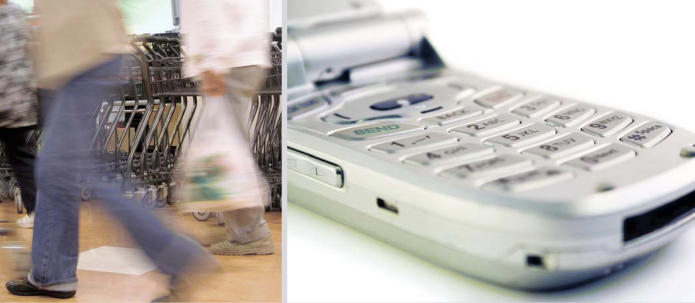
Phoning in the facts New sensor technologies will make use of cell phones and similar apparatus to continuously monitor individuals as they go about daily life, recording data on physical activity, environmental exposures, and measures such as heart rate and respiration.

**Figure:**
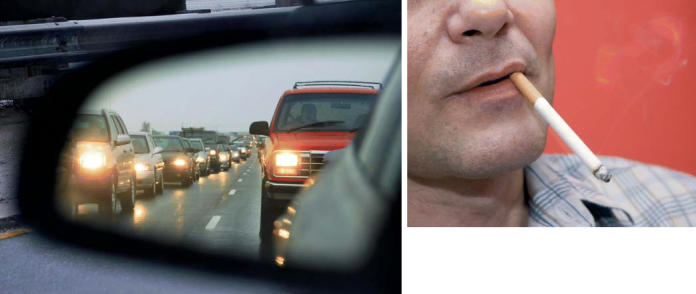
Making the connection A major goal of exposure biology is to identify specific biomarkers of exposure to agents such as vehicular air pollution and environmental tobacco smoke, then link them to mechanisms of disease.

